# Crystal structure of 2-amino-4,6-di­meth­oxy­pyrimidinium thio­phene-2-carboxyl­ate

**DOI:** 10.1107/S2056989015010907

**Published:** 2015-06-13

**Authors:** Ammaiyappan Rajam, P.T. Muthiah, Ray J. Butcher, Jerry P. Jasinski

**Affiliations:** a School of Chemistry, Bharathidasan University, Tiruchirappalli 620 024, Tamilnadu, India; b Department of Chemistry, Howard University, 525 College Street NW, Washington, DC 20059, USA; c Department of Chemistry, Keene State College, 229 Main Street, Keene, NH 03435-2001, USA

**Keywords:** crystal structure, crystal salts, hydrogen-bonding patterns, π–π stacking inter­actions

## Abstract

In the title salt, C_6_H_10_N_3_O_2_^+^·C_5_H_3_O_2_S^−^, the 2-amino-4,6-di­meth­oxy­pyrimidinium cation inter­acts with the carboxyl­ate group of the thio­phene-2-carboxyl­ate anion through a pair of N—H⋯O hydrogen bonds, forming an *R*_2_^2^(8) ring motif. These motifs are centrosymmetrically paired *via* N—H⋯O hydrogen bonds, forming a complementary *DDAA* array. The separate *DDAA* arrays are linked by π–π stacking inter­actions between the pyrimidine rings, as well as by a number of weak C—H⋯O and N—H⋯O inter­actions. In the anion, the dihedral angle between the ring plane and the CO_2_ group is 11.60 (3)°. In the cation, the C atoms of methoxy groups deviate from the ring plane by 0.433 (10) Å.

## Related literature   

For the role played by non-covalent inter­actions in mol­ecular recognition porcesses, see: Desiraju (1989 ▸). For amino­pryimidine–carboxylate interactions in protein–nucleic acid recognition and protein–drug binding inteactions, see: Hunt *et al.* (1980 ▸); Alkorta & Elguero (2003 ▸). For 1:1 salts between 2-amino­pyrimidine and mono- and di­carb­oxy­lic acids, see: Etter & Adsmond (1990 ▸). For self-assembly of 2-amino­pyrimidine compounds, see: Scheinbeim & Schempp (1976 ▸). For carb­oxy­lic acid and 2-amino heterocyclic ring system synthons, see: Lynch & Jones (2004 ▸). For crystal structures of related salts, see: Ebenezer *et al.* (2012 ▸); Jennifer & Mu­thiah (2014 ▸). *DDAA* arrays have been observed in trimeth­oprim hydrogen glutarate (Robert *et al.*, 2001 ▸), trimetho­prim formate (Umadevi *et al.*, 2002 ▸), trimethoprim-*m*-chloro­benzoate (Raj *et al.*, 2003 ▸), pyrimethaminium 3,5-di­nitro­benzoate (Subashini *et al.*, 2007 ▸) and 2-amino-4,6-di­meth­oxy­pyrimidinum-salicylate (Thanigaimani *et al.*, 2007 ▸).
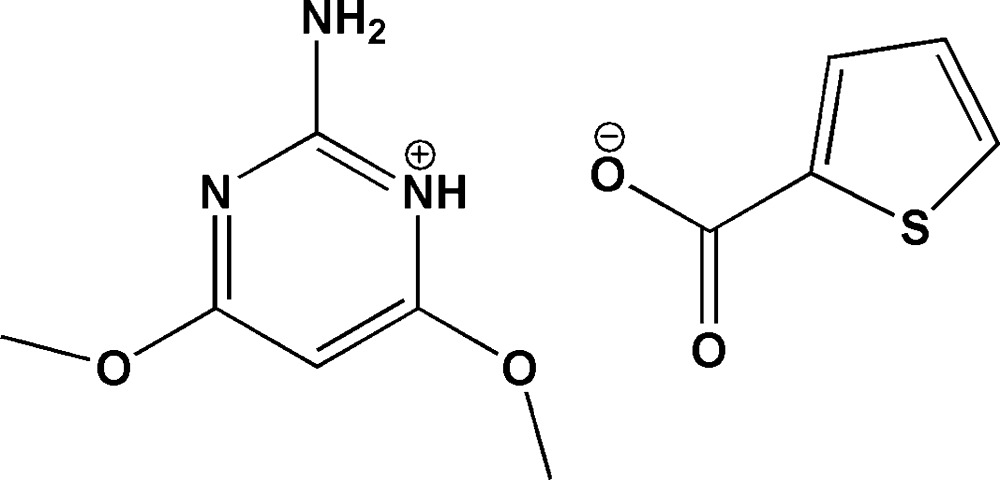


## Experimental   

### Crystal data  

C_6_H_10_N_3_O_2_^+^·C_5_H_3_O_2_S^−^
*M* *_r_* = 283.30
Monoclinic, 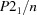

*a* = 6.7335 (3) Å
*b* = 7.6307 (4) Å
*c* = 25.0638 (10) Å
β = 93.928 (4)°
*V* = 1284.78 (10) Å^3^
*Z* = 4
Mo *K*α radiation
μ = 0.27 mm^−1^
*T* = 173 K
0.32 × 0.28 × 0.14 mm

### Data collection  

Agilent Eos Gemini diffractometer
Absorption correction: multi-scan (*CrysAlis RED*; Agilent, 2012 ▸) *T*_min_ = 0.789, *T*_max_ = 1.000
8844 measured reflections
4245 independent reflections
3071 reflections with *I* > 2σ(*I*)
*R*_int_ = 0.028

### Refinement  

*R*[*F* ^2^ > 2σ(*F* ^2^)] = 0.062
*wR*(*F* ^2^) = 0.186
*S* = 1.05
4245 reflections
174 parameters
H-atom parameters constrained
Δρ_max_ = 0.79 e Å^−3^
Δρ_min_ = −0.55 e Å^−3^

### 

Data collection: *CrysAlis PRO* (Agilent, 2012 ▸); cell refinement: *CrysAlis PRO*; data reduction: *CrysAlis RED* (Agilent, 2012 ▸); program(s) used to solve structure: *SUPERFLIP* (Palatinus & Chapuis, 2007 ▸; Palatinus & van der Lee, 2008 ▸; Palatinus *et al.*, 2012 ▸); program(s) used to refine structure: *SHELXL2014* (Sheldrick, 2015 ▸); molecular graphics: *OLEX2* (Dolomanov *et al.*, 2009 ▸); software used to prepare material for publication: *OLEX2*.

## Supplementary Material

Crystal structure: contains datablock(s) global, I. DOI: 10.1107/S2056989015010907/hg5442sup1.cif

Structure factors: contains datablock(s) I. DOI: 10.1107/S2056989015010907/hg5442Isup2.hkl

Supporting information file. DOI: 10.1107/S2056989015010907/hg5442Isup3.cml

**Fig. 1.**
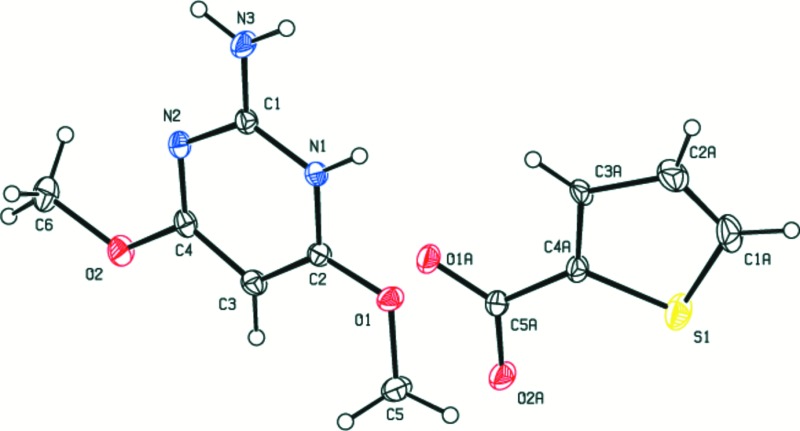
: The asymmetric unit of the title compound, showing 30% probability displacement ellipsoids.

**Fig. 2.**
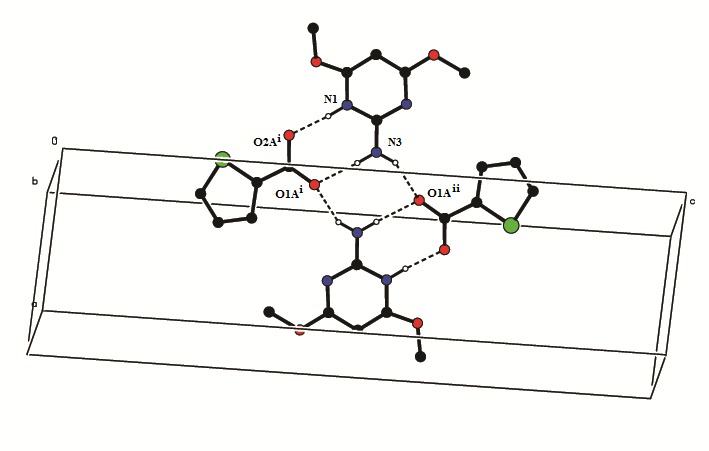
: A view of DDAA array along the b axis formed by independent N—H···O hydrogen bonds. Symmetry codes are given in Table 1. Dashed lines represent hydrogen bonds.

. DOI: 10.1107/S2056989015010907/hg5442fig1.tif
The asymmetric unit of the title compound, showing 30% probability displacement ellipsoids.

b . DOI: 10.1107/S2056989015010907/hg5442fig2.tif
A view of DDAA array along the *b* axis formed by independent N—H⋯O hydrogen bonds. Symmetry codes are given in Table 1. Dashed lines represent hydrogen bonds.

**Fig. 3.**
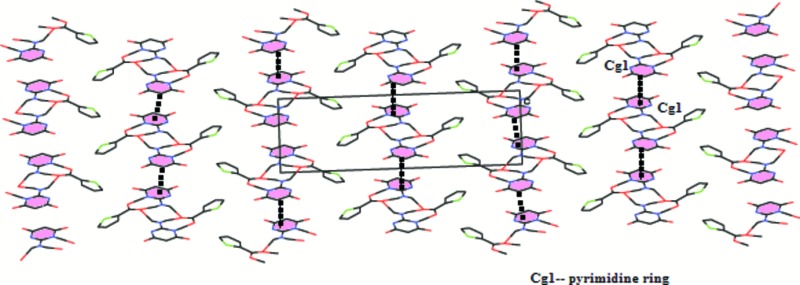
: A view of infinite number of DDAA arrays interconnected by π–π stacking interactions indicated by dotted lines. Cg1···Cg1 = 3.4689 (12) Å, where Cg1 represents the centroid of the ring N1/C1/N2/C2/C3/C4.

Cg Cg Cg . DOI: 10.1107/S2056989015010907/hg5442fig3.tif
A view of infinite number of DDAA arrays inter­connected by π–π stacking inter­actions indicated by dotted lines. *Cg*1⋯*Cg*1 = 3.4689 (12) Å, where *Cg*1 represents the centroid of the ring N1/C1/N2/C2/C3/C4.

CCDC reference: 1405154

Additional supporting information:  crystallographic information; 3D view; checkCIF report

## Figures and Tables

**Table 1:** Hydrogen-bond geometry (, )

*D*H*A*	*D*H	H*A*	*D* *A*	*D*H*A*
N1H1O2*A* ^i^	0.88	1.76	2.637(2)	175
N3H3*A*O1*A* ^ii^	0.88	2.04	2.826(2)	148
N3H3*B*O1*A* ^i^	0.88	1.92	2.798(2)	173
C6H6*B*O1^iii^	0.98	2.52	3.434(3)	155
C1*A*H1*A*O1^iv^	0.95	2.60	3.365(3)	138
C1*A*H1*A*O2*A* ^v^	0.95	2.60	3.383(3)	140

Symmetry codes: (i) 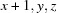; (ii) 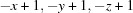; (iii) 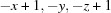; (iv) 
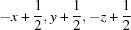
; (v) 
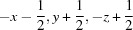
.

## supplementary crystallographic information

### S1. Structural commentary

The asymmetric unit of C_6_H_10_N_3_O_2_^+^ C_5_H_3_O_2_S^-^, (I), contains one 2-amino-4,6-di­meth­oxy­pyrimidinium cation and one thio­phene-2-carboxyl­ate anion (Fig 1). Protonation of the cation occurs at N1, providing a C1/N1/C2 angle of 119.39 (16)° compared to the C1/N2/C4 angle (115.99 (16)°) of the unprotonated N2 atom. The carboxyl­ate group of the thio­phene-2-carboxyl­ate anion inter­acts with the protonated atom N1 and the 2-amino group of the pyrimidine moiety through a pair of N—H···O hydrogen bonds, forming an eight membered *R*^2^_2_(8) ring motif. These motifs are centrosymmetrically paired via N—H···O hydrogen bonds to produce a DDAA (D = donor in hydrogen bonds, A = acceptor in hydrogen bonds) array of quadruple hydrogen bonds represented by the graph-set notation *R*^2^_2_(8), *R*^4^_2_(8) and *R*^2^_2_(8) (Fig. 2). This type of array has also been identified in trimethoprim hydrogen glutarate (Robert *et al.*, 2001), trimethoprim formate (Umadevi *et al.*, 2002), trimethoprim- m-chloro­benzoate (Raj *et al.*, 2003), pyrimethaminium 3,5-di­nitro­benzoate (Subashini *et al.*, 2007) and 2-amino-4,6-di­meth­oxy­pyrimidinum-salicylate (Thanigaimani *et al.*, 2007). An infinite number of several such quadruple arrays are inter­connected and stabilized by π—π stacking inter­actions between the pyrimidine ring of one array with a neighbouring array, with an observed inter­planar distance of 3.356 Å, a centroid (Cg1)-to-centroid (Cg1) distance of 3.4689 (12) Å (where Cg1 equals the centroid of the ring N1/C1/N2/C2/C3/C4, Fig 3) and slip angle (the angle between the centroid vector and the normal to the plane) of 14.68°, which are typical aromatic stacking values (Hunter, 1994). In addition, a number of weak C—H···O and N—H···O inter­molecular inter­actions are also observed which contribute to crystal packing stability (Table 2).

### S2. Synthesis and crystallization

A hot methano­lic solution of 2-amino-4,6-di­meth­oxy pyrimidine (38 mg, Aldrich) and thio­phene-2-carb­oxy­lic acid (32 mg, Aldrich) was warmed for half an hour over a water bath. The mixture was cooled slowly and kept at room temperature. After a few days colourless crystals were obtained.

### S3. Refinement

Crystal data, data collection and structure refinement details are summarized in Table 1. All of the H atoms were placed in their calculated positions andthen refined using the riding model with atom—H lengths of 0.95Å (CH), 0.98Å (CH_3_)or 0.88Å (NH, NH_2_). Isotropic displacement parameters for these atoms were set to 1.2 (CH, NH, NH_2_) or 1.5 (CH_3_) times Ueq of the parent atom. Idealised Me refined as rotating groups.

### Crystal data

**Table:**

C_6_H_10_N_3_O_2_^+^·C_5_H_3_O_2_S^−^	*F*(000) = 592
*M**_r_* = 283.30	*D*_x_ = 1.465 Mg m^−^^3^
Monoclinic, *P*2_1_/*n*	Mo *K*α radiation, λ = 0.71073 Å
*a* = 6.7335 (3) Å	Cell parameters from 2092 reflections
*b* = 7.6307 (4) Å	θ = 4.0–32.4°
*c* = 25.0638 (10) Å	µ = 0.27 mm^−^^1^
β = 93.928 (4)°	*T* = 173 K
*V* = 1284.78 (10) Å^3^	Irregular, colourless
*Z* = 4	0.32 × 0.28 × 0.14 mm

### Data collection

**Table:**

Agilent Eos Gemini diffractometer	4245 independent reflections
Radiation source: Enhance (Mo) X-ray Source	3071 reflections with *I* > 2σ(*I*)
Detector resolution: 16.0416 pixels mm^-1^	*R*_int_ = 0.028
ω scans	θ_max_ = 32.7°, θ_min_ = 3.3°
Absorption correction: multi-scan (*CrysAlis RED*; Agilent, 2012)	*h* = −7→9
*T*_min_ = 0.789, *T*_max_ = 1.000	*k* = −11→10
8844 measured reflections	*l* = −36→33

### Refinement

**Table:**

Refinement on *F*^2^	Primary atom site location: structure-invariant direct methods
Least-squares matrix: full	Hydrogen site location: inferred from neighbouring sites
*R*[*F*^2^ > 2σ(*F*^2^)] = 0.062	H-atom parameters constrained
*wR*(*F*^2^) = 0.186	*w* = 1/[σ^2^(*F*_o_^2^) + (0.0861*P*)^2^ + 0.9218*P*] where *P* = (*F*_o_^2^ + 2*F*_c_^2^)/3
*S* = 1.05	(Δ/σ)_max_ < 0.001
4245 reflections	Δρ_max_ = 0.79 e Å^−^^3^
174 parameters	Δρ_min_ = −0.55 e Å^−^^3^
0 restraints	

### Special details

**Table:**

Geometry. All e.s.d.'s (except the e.s.d. in the dihedral angle between two l.s. planes) are estimated using the full covariance matrix. The cell e.s.d.'s are taken into account individually in the estimation of e.s.d.'s in distances, angles and torsion angles; correlations between e.s.d.'s in cell parameters are only used when they are defined by crystal symmetry. An approximate (isotropic) treatment of cell e.s.d.'s is used for estimating e.s.d.'s involving l.s. planes.

### Fractional atomic coordinates and isotropic or equivalent isotropic displacement parameters (Å^2^)

**Table:**

	*x*	*y*	*z*	*U*_iso_*/*U*_eq_	
O1	0.3208 (2)	0.1426 (2)	0.38775 (6)	0.0280 (3)	
O2	0.2312 (2)	0.1346 (2)	0.57319 (6)	0.0324 (4)	
N1	0.5394 (2)	0.2619 (2)	0.44749 (6)	0.0228 (3)	
H1	0.6074	0.2925	0.4202	0.027*	
N2	0.5086 (2)	0.2608 (2)	0.54146 (6)	0.0240 (3)	
N3	0.7832 (3)	0.3787 (3)	0.50524 (7)	0.0329 (4)	
H3A	0.8323	0.4043	0.5377	0.039*	
H3B	0.8504	0.4053	0.4775	0.039*	
C1	0.6093 (3)	0.3005 (3)	0.49820 (8)	0.0230 (4)	
C2	0.3645 (3)	0.1761 (3)	0.43926 (8)	0.0223 (4)	
C3	0.2549 (3)	0.1307 (3)	0.48120 (8)	0.0257 (4)	
H3	0.1315	0.0706	0.4763	0.031*	
C4	0.3372 (3)	0.1789 (3)	0.53174 (8)	0.0242 (4)	
C5	0.1469 (3)	0.0367 (3)	0.37450 (9)	0.0333 (5)	
H5A	0.1320	0.0186	0.3357	0.050*	
H5B	0.0286	0.0966	0.3862	0.050*	
H5C	0.1617	−0.0769	0.3926	0.050*	
C6	0.3184 (4)	0.1689 (4)	0.62628 (9)	0.0352 (5)	
H6A	0.3659	0.2903	0.6284	0.053*	
H6B	0.4305	0.0890	0.6342	0.053*	
H6C	0.2182	0.1507	0.6523	0.053*	
S1	−0.15194 (10)	0.54563 (10)	0.26469 (2)	0.0427 (2)	
O1A	−0.0066 (2)	0.4332 (2)	0.41417 (6)	0.0326 (4)	
O2A	−0.2775 (2)	0.3607 (3)	0.36279 (6)	0.0361 (4)	
C1A	0.0377 (5)	0.6516 (4)	0.23842 (10)	0.0434 (6)	
H1A	0.0295	0.7014	0.2036	0.052*	
C2A	0.2037 (4)	0.6582 (4)	0.27227 (11)	0.0431 (6)	
H2A	0.3227	0.7142	0.2631	0.052*	
C3A	0.1860 (3)	0.5737 (3)	0.32322 (8)	0.0268 (4)	
H3AA	0.2874	0.5637	0.3513	0.032*	
C4A	−0.0153 (3)	0.5071 (3)	0.32345 (8)	0.0253 (4)	
C5A	−0.1048 (3)	0.4276 (3)	0.37009 (8)	0.0250 (4)	

### Atomic displacement parameters (Å^2^)

**Table:**

	*U*^11^	*U*^22^	*U*^33^	*U*^12^	*U*^13^	*U*^23^
O1	0.0286 (7)	0.0348 (8)	0.0209 (7)	−0.0082 (6)	0.0042 (5)	−0.0050 (6)
O2	0.0321 (8)	0.0437 (10)	0.0228 (7)	−0.0045 (7)	0.0110 (6)	0.0022 (6)
N1	0.0241 (7)	0.0250 (8)	0.0196 (7)	−0.0027 (6)	0.0044 (6)	0.0006 (6)
N2	0.0269 (8)	0.0271 (8)	0.0185 (7)	0.0015 (6)	0.0054 (6)	0.0015 (6)
N3	0.0316 (9)	0.0479 (12)	0.0193 (8)	−0.0135 (8)	0.0023 (7)	−0.0005 (8)
C1	0.0267 (9)	0.0229 (9)	0.0198 (8)	0.0003 (7)	0.0039 (7)	0.0016 (7)
C2	0.0241 (9)	0.0214 (9)	0.0217 (8)	−0.0009 (7)	0.0032 (7)	−0.0006 (7)
C3	0.0241 (9)	0.0279 (10)	0.0256 (9)	−0.0034 (7)	0.0062 (7)	0.0015 (8)
C4	0.0275 (9)	0.0243 (9)	0.0217 (9)	0.0032 (7)	0.0079 (7)	0.0029 (7)
C5	0.0321 (10)	0.0379 (12)	0.0296 (11)	−0.0087 (9)	0.0009 (8)	−0.0066 (9)
C6	0.0372 (11)	0.0494 (14)	0.0202 (9)	0.0037 (10)	0.0101 (8)	−0.0003 (9)
S1	0.0476 (4)	0.0578 (5)	0.0226 (3)	0.0000 (3)	0.0015 (2)	0.0018 (3)
O1A	0.0325 (8)	0.0457 (10)	0.0194 (7)	−0.0082 (7)	−0.0006 (6)	0.0043 (6)
O2A	0.0340 (8)	0.0530 (11)	0.0213 (7)	−0.0147 (7)	0.0015 (6)	0.0006 (7)
C1A	0.0661 (17)	0.0416 (14)	0.0241 (11)	0.0038 (12)	0.0141 (11)	0.0050 (10)
C2A	0.0517 (15)	0.0439 (15)	0.0354 (13)	−0.0110 (11)	0.0155 (11)	0.0029 (11)
C3A	0.0409 (11)	0.0239 (9)	0.0170 (8)	−0.0072 (8)	0.0112 (7)	0.0013 (7)
C4A	0.0311 (10)	0.0277 (10)	0.0171 (8)	0.0014 (8)	0.0026 (7)	0.0002 (7)
C5A	0.0291 (9)	0.0277 (10)	0.0186 (8)	−0.0026 (7)	0.0034 (7)	−0.0002 (7)

### Geometric parameters (Å, º)

**Table:**

O1—C2	1.329 (2)	C5—H5B	0.9800
O1—C5	1.443 (3)	C5—H5C	0.9800
O2—C4	1.343 (2)	C6—H6A	0.9800
O2—C6	1.441 (3)	C6—H6B	0.9800
N1—H1	0.8800	C6—H6C	0.9800
N1—C1	1.357 (2)	S1—C1A	1.683 (3)
N1—C2	1.351 (3)	S1—C4A	1.708 (2)
N2—C1	1.352 (2)	O1A—C5A	1.249 (2)
N2—C4	1.321 (3)	O2A—C5A	1.272 (3)
N3—H3A	0.8800	C1A—H1A	0.9500
N3—H3B	0.8800	C1A—C2A	1.357 (4)
N3—C1	1.315 (3)	C2A—H2A	0.9500
C2—C3	1.370 (3)	C2A—C3A	1.443 (3)
C3—H3	0.9500	C3A—H3AA	0.9500
C3—C4	1.396 (3)	C3A—C4A	1.448 (3)
C5—H5A	0.9800	C4A—C5A	1.481 (3)
			
C2—O1—C5	116.97 (16)	H5A—C5—H5C	109.5
C4—O2—C6	117.66 (17)	H5B—C5—H5C	109.5
C1—N1—H1	120.3	O2—C6—H6A	109.5
C2—N1—H1	120.3	O2—C6—H6B	109.5
C2—N1—C1	119.40 (16)	O2—C6—H6C	109.5
C4—N2—C1	115.98 (17)	H6A—C6—H6B	109.5
H3A—N3—H3B	120.0	H6A—C6—H6C	109.5
C1—N3—H3A	120.0	H6B—C6—H6C	109.5
C1—N3—H3B	120.0	C1A—S1—C4A	92.37 (12)
N2—C1—N1	122.80 (18)	S1—C1A—H1A	123.6
N3—C1—N1	118.20 (17)	C2A—C1A—S1	112.83 (19)
N3—C1—N2	119.00 (18)	C2A—C1A—H1A	123.6
O1—C2—N1	111.98 (16)	C1A—C2A—H2A	122.5
O1—C2—C3	126.99 (18)	C1A—C2A—C3A	115.0 (2)
N1—C2—C3	121.02 (18)	C3A—C2A—H2A	122.5
C2—C3—H3	122.3	C2A—C3A—H3AA	126.4
C2—C3—C4	115.38 (18)	C2A—C3A—C4A	107.1 (2)
C4—C3—H3	122.3	C4A—C3A—H3AA	126.4
O2—C4—C3	115.91 (18)	C3A—C4A—S1	112.68 (14)
N2—C4—O2	118.68 (18)	C3A—C4A—C5A	125.34 (18)
N2—C4—C3	125.40 (17)	C5A—C4A—S1	121.78 (16)
O1—C5—H5A	109.5	O1A—C5A—O2A	124.37 (18)
O1—C5—H5B	109.5	O1A—C5A—C4A	117.69 (18)
O1—C5—H5C	109.5	O2A—C5A—C4A	117.92 (18)
H5A—C5—H5B	109.5		
			
O1—C2—C3—C4	178.67 (19)	C6—O2—C4—N2	−4.2 (3)
N1—C2—C3—C4	−0.2 (3)	C6—O2—C4—C3	174.98 (19)
C1—N1—C2—O1	−177.64 (17)	S1—C1A—C2A—C3A	0.3 (3)
C1—N1—C2—C3	1.3 (3)	S1—C4A—C5A—O1A	−167.06 (17)
C1—N2—C4—O2	179.44 (18)	S1—C4A—C5A—O2A	11.8 (3)
C1—N2—C4—C3	0.4 (3)	C1A—S1—C4A—C3A	−1.39 (18)
C2—N1—C1—N2	−1.8 (3)	C1A—S1—C4A—C5A	173.67 (19)
C2—N1—C1—N3	177.7 (2)	C1A—C2A—C3A—C4A	−1.3 (3)
C2—C3—C4—O2	−179.84 (19)	C2A—C3A—C4A—S1	1.7 (2)
C2—C3—C4—N2	−0.7 (3)	C2A—C3A—C4A—C5A	−173.1 (2)
C4—N2—C1—N1	0.9 (3)	C3A—C4A—C5A—O1A	7.3 (3)
C4—N2—C1—N3	−178.5 (2)	C3A—C4A—C5A—O2A	−173.8 (2)
C5—O1—C2—N1	174.26 (18)	C4A—S1—C1A—C2A	0.6 (2)
C5—O1—C2—C3	−4.7 (3)		

### Hydrogen-bond geometry (Å, º)

**Table:**

*D*—H···*A*	*D*—H	H···*A*	*D*···*A*	*D*—H···*A*
N1—H1···O1*A*^i^	0.88	2.83	3.479 (2)	132
N1—H1···O2*A*^i^	0.88	1.76	2.637 (2)	175
N3—H3*A*···O1*A*^ii^	0.88	2.04	2.826 (2)	148
N3—H3*B*···O1*A*^i^	0.88	1.92	2.798 (2)	173
C5—H5*B*···O1*A*	0.98	2.68	3.369 (3)	128
C6—H6*B*···O1^iii^	0.98	2.52	3.434 (3)	155
C1*A*—H1*A*···O1^iv^	0.95	2.60	3.365 (3)	138
C1*A*—H1*A*···O2*A*^v^	0.95	2.60	3.383 (3)	140

Symmetry codes: (i) *x*+1, *y*, *z*; (ii) −*x*+1, −*y*+1, −*z*+1; (iii) −*x*+1, −*y*, −*z*+1; (iv) −*x*+1/2, *y*+1/2, −*z*+1/2; (v) −*x*−1/2, *y*+1/2, −*z*+1/2.

## References

[bb1] Agilent (2012). *CrysAlis PRO* and *CrysAlis RED*. Agilent Technologies Ltd, Yarnton, England.

[bb2] Alkorta, I. & Elguero, J. (2003). *J. Phys. Chem.* **107**, 5306–5310.

[bb4] Desiraju, G. R. (1989). In *Crystal Engineering: The Design of Organic Solids*. Amsterdam: Elsevier.

[bb5] Dolomanov, O. V., Bourhis, L. J., Gildea, R. J., Howard, J. A. K. & Puschmann, H. (2009). *J. Appl. Cryst.* **42**, 339–341.

[bb6] Ebenezer, S. & Muthiah, P. T. (2012). *Cryst. Growth Des.* **12**, 3766–3785.

[bb7] Etter, M. C. & Adsmond, D. A. (1990). *J. Chem. Soc. Chem. Commun.* pp. 589–591.

[bb8] Hunt, W. E., Schwalbe, C. H., Bird, K. & Mallinson, P. D. (1980). *Biochem. J.* **187**, 533–536. 10.1042/bj1870533 PMC1161822 6893149

[bb10] Jennifer, S. J. & Muthiah, P. T. (2014). *Chem. Cent. J.* **8**, 20. 10.1186/1752-153X-8-20 PMC3996520 24655545

[bb11] Lynch, D. E. & Jones, G. D. (2004). *Acta Cryst.* B**60**, 748–754. 10.1107/S0108768104023791 15534386

[bb12] Palatinus, L. & Chapuis, G. (2007). *J. Appl. Cryst.* **40**, 786–790.

[bb13] Palatinus, L., Prathapa, S. J. & van Smaalen, S. (2012). *J. Appl. Cryst.* **45**, 575–580.

[bb14] Palatinus, L. & van der Lee, A. (2008). *J. Appl. Cryst.* **41**, 975–984.

[bb3] Raj, S. B., Muthiah, P. T., Rychlewska, U. & Warzajtis, B. (2003). *CrystEngComm.*, **5**, 48–53.

[bb15] Robert, J. J., Raj, S. B. & Muthiah, P. T. (2001). *Acta Cryst.* E**57**, o1206–o1208.

[bb16] Scheinbeim, J. & Schempp, E. (1976). *Acta Cryst.* B**32**, 607–609.

[bb17] Sheldrick, G. M. (2015). *Acta Cryst.* C**71**, 3–8.

[bb18] Subashini, A., Muthiah, P. T., Bocelli, G. & Cantoni, A. (2007). *Acta Cryst.* E**63**, o3775. 10.1107/S1600536807067190 PMC2915166 21200590

[bb19] Thanigaimani, K., Muthiah, P. T. & Lynch, D. E. (2007). *Acta Cryst.* E**63**, o4555–o4556. 10.1107/S0108270107005677 17478916

[bb20] Umadevi, B., Prabakaran, P. & Muthiah, P. T. (2002). *Acta Cryst.* C**58**, o510–o512. 10.1107/s0108270102011150 12154314

