# Supplementary figures and images for: Crystal structure of 2-amino-4,6-di­meth­oxy­pyrimidinium thio­phene-2-carboxyl­ate
Source: Acta Crystallogr E Crystallogr Commun. 2015 Jun 13;71(Pt 7):o479–80. doi: 10.1107/S2056989015010907 (PMC4518905; doi:10.1107/S2056989015010907)

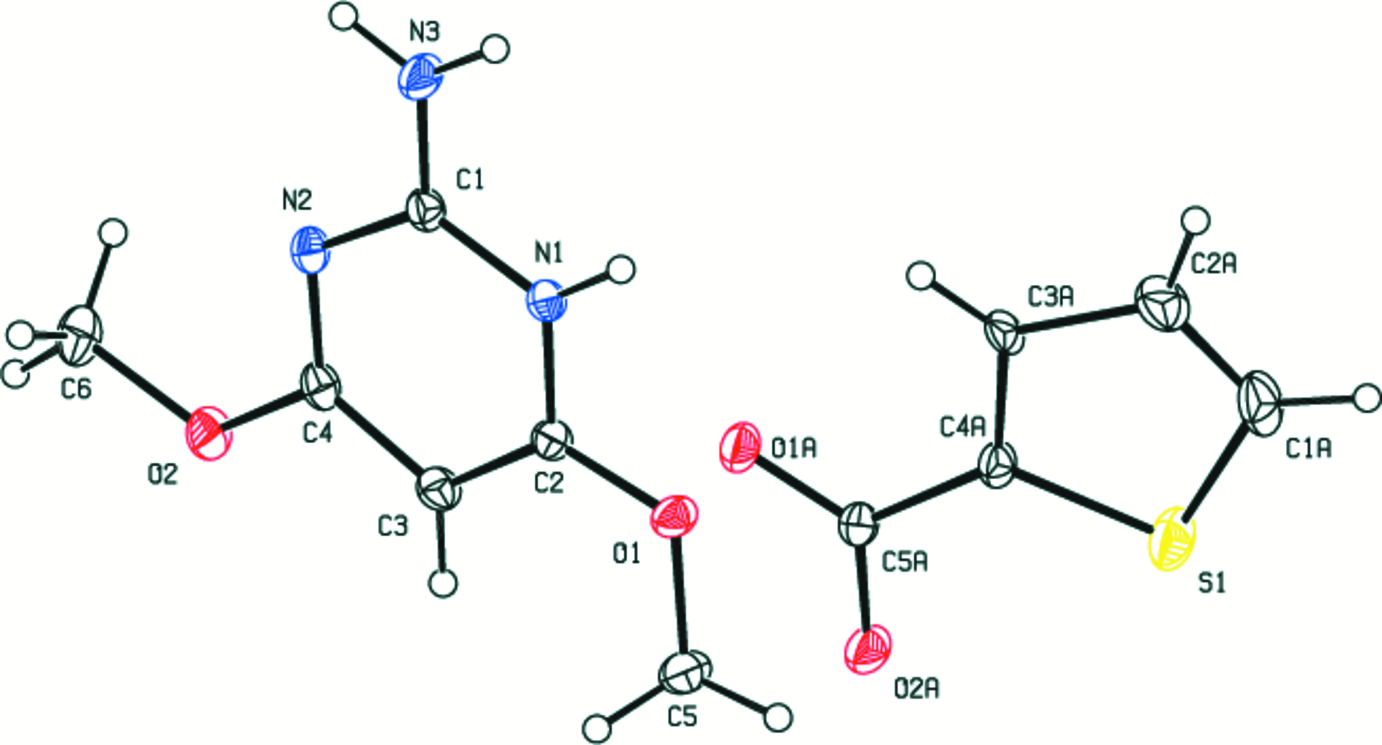

Supplement: Supplementary file 4 [file e-71-0o479-fig1.tif]

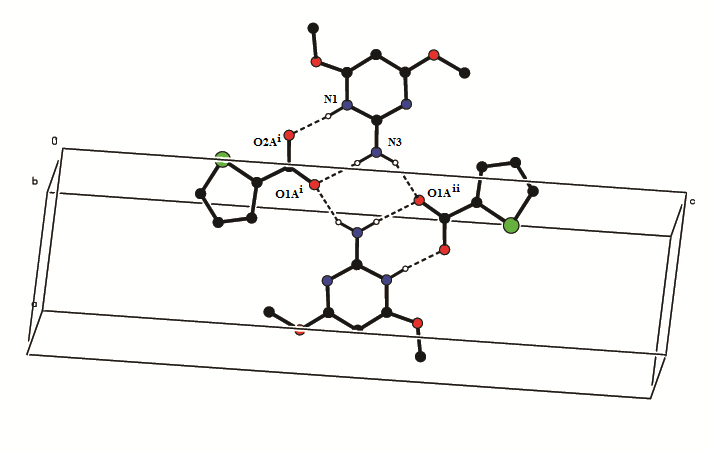

Supplement: Supplementary file 5 [file e-71-0o479-fig2.tif]

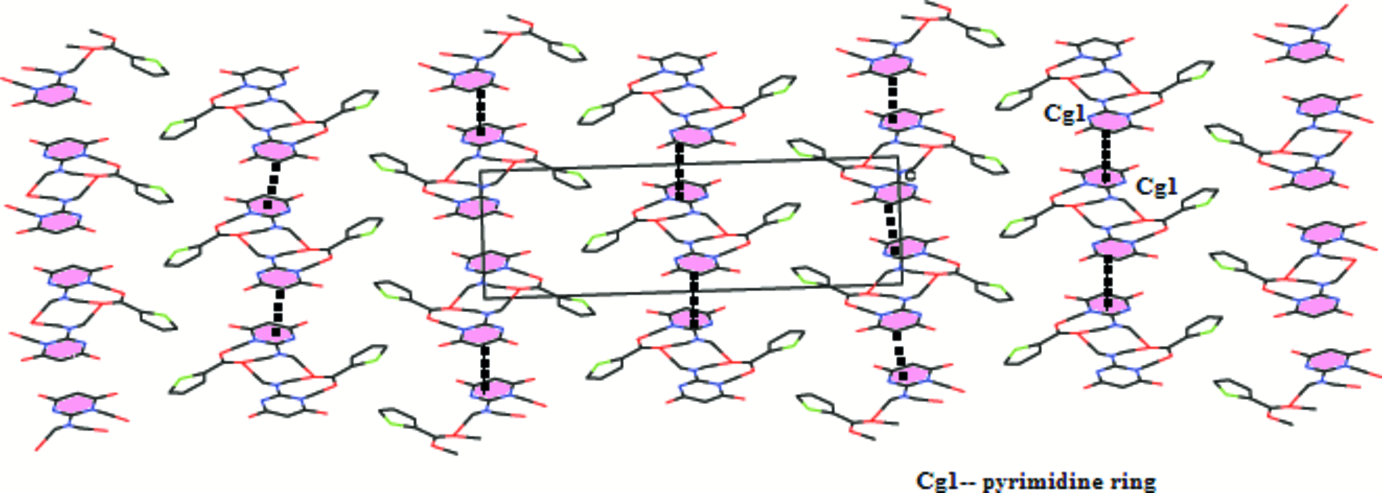

Supplement: Supplementary file 6 [file e-71-0o479-fig3.tif]
